# Editorial: The Effect of Carbohydrate Restriction on Cancer and Metabolic Syndrome

**DOI:** 10.3389/fnut.2022.823302

**Published:** 2022-02-14

**Authors:** Ingrid Elisia, Sylvia Santosa, David G. Popovich, Gerald Krystal

**Affiliations:** ^1^Terry Fox Laboratory, British Columbia Cancer Research Centre, Vancouver, BC, Canada; ^2^Department of Health, Kinesiology and Applied Physiology, Concordia University, Montreal, QC, Canada; ^3^School of Food and Advanced Technology, Massey University, Palmerston North, New Zealand

**Keywords:** cancer, sugar, ketogenic, low carbohydrate diet, metabolic syndrome

Currently, low carbohydrate (LC) diets, including ketogenic diets (KDs), are not only gaining popularity for weight loss but are also being investigated for the prevention and treatment of cancer, diabetes, and metabolic syndrome (1–3). In this special issue, we present six articles evaluating the safety/efficacy of LC/KDs in the prevention and treatment of cancer and metabolic syndrome.

Using glucose restriction to prevent and treat cancer is based on the observation that cancer cells typically consume and require more glucose than normal cells (the Warburg effect) (4). Currently, data supporting the safety and efficacy of a LC/KD for the prevention/treatment of cancer are limited to pre-clinical animal studies and a few case reports. Herein, we present a systematic review and meta-analysis of animal studies by Li et al. which point to an overall anti-tumor effect of KDs, albeit with a limited number of tumor types. However, there is a lack of high-quality data in humans because no randomized placebo-controlled double-blind clinical trials have been carried out in this area. Nevertheless, the online survey results in the Tulipan and Kofler article suggest that LC diets improve the quality of life of cancer patients and normalize their body weight. The survey results also indicate that some cancer patients adopt a KD or LC diet on their own without professional counseling and without informing their doctors.

Sukkar and Muscaritoli discuss the various dietary regimens being used currently by cancer patients and the scientific rationale for their use. They stress the importance of cancer patients receiving medical supervision when adopting a LC diet since these diets may affect their wellbeing during cancer treatment, particularly since there are many types of LC diets. They find that some types of LC diets may lead to vitamin/mineral deficiencies and unwanted weight loss that may be detrimental for cancer patients. Since clinical evidence demonstrating the safety and efficacy of LC diets as an adjunct for cancer patients is currently lacking, there is a gap between the advice that can be given by medical/health care workers and the professional advice that cancer patients need. Related to this, Haskins et al. provide a thorough review of traditional cancer therapies and possible synergies with LC diets. Given that there are many ongoing clinical trials investigating the safety and efficacy of LC/KDs in combination with various cancer therapies, this gap could be filled in the not too distant future.

The main mechanism by which a KD is thought to elicit clinical benefits is by inducing a metabolic switch that pushes the body to utilize fats *via* ketogenesis rather than glucose as an energy source. During ketogenesis, fats are broken down to generate ketone bodies, which can then fuel oxidative phosphorylation in normal cells for ATP generation. Cancer cells are thought to lack one or more of the ketolytic enzymes necessary to break down ketone bodies for energy, thus leading to their selective death when patients are on a KD. The observation that the health benefits of a KD typically correlate with an increase in ketone bodies has given rise to the use of ketone body supplementation alone to evoke a comparable clinical impact as a KD. However, Buga et al. suggest that while a KD favorably impacts body composition, ketone salts themselves have no impact, suggesting the importance of a whole body metabolic switch and not just the elevation of ketone bodies in producing health benefits.

Lastly, Elisia and Krystal highlight the fact that many KDs are high in saturated fats, known to be pro-inflammatory (5–7). Thus, some KDs may contribute to chronic inflammation and, in turn, cancer development (8). Other fats, like unsaturated omega 3 fatty acids have anti-inflammatory properties (5, 9), and so may reduce cancer development. The authors therefore encourage future studies to indicate the types of fats used. Also, since not all cancers rely on glucose [e.g., early stage prostate cancer appears to prefer fatty acids as a nutrient source (10)], a LC/KD may also not be effective for all types of cancer. Elisia and Krystal also report that the short-chain fatty acids (SCFAs) generated from the fermentation of soluble fiber and resistant starch by the gut microbiome are more potently anti-inflammatory than beta-hydroxybutyrate—an important ketone body produced when one adopts a LC/KD. They suggest that one of the limitations of a LC/KD is that it is typically deficient in soluble fiber/resistant starch and thus results in low levels of anti-inflammatory SCFAs. Whether a LC/KD can be optimized so that soluble fiber/resistant starch can be incorporated is another research question warranting further investigation.

To conclude, while LC/KDs have given promising results to date, there is still more research required before a medical professional can advise whether a LC/KD may offer health benefits in the treatment/prevention of specific cancers and/or metabolic syndrome. Considering that there is a clear interest in LC/KDs for metabolic syndrome and as a cancer adjunct, future research should aim to provide evidence of efficacy against tumors with different metabolic requirements as well as define the role of different fat types in LC/KDs on inflammatory status.

## Author Contributions

IE wrote the first draft. SS, DP, and GK edited and approved the final draft. All authors contributed to the article and approved the submitted version.

## Funding

The research was supported by the Lotte and John Hecht Memorial Foundation (#4340), with core support from the BC Cancer Foundation and BC Cancer.

## Conflict of Interest

The authors declare that the research was conducted in the absence of any commercial or financial relationships that could be construed as a potential conflict of interest.

## Publisher's Note

All claims expressed in this article are solely those of the authors and do not necessarily represent those of their affiliated organizations, or those of the publisher, the editors and the reviewers. Any product that may be evaluated in this article, or claim that may be made by its manufacturer, is not guaranteed or endorsed by the publisher.
